# Cognitive impairment in Sjogren’s syndrome: Interplay between BACE1 activity, inflammatory blood biomarkers and neurocognitive testing

**DOI:** 10.1371/journal.pone.0328311

**Published:** 2025-08-01

**Authors:** Alireza Kooshki, Mohammad Yousefi, Reyhane Farmani, Hamid Kabiri-Rad, Hossien Nezami, Amirhosein Zardast, Zeinab Saremi

**Affiliations:** 1 Student Research Committee, Birjand University of Medical Sciences, Birjand, Iran; 2 Cellular and Molecular Research Center, Birjand University of Medical Sciences, Birjand, Iran; 3 Department of Epidemiology and Biostatistics, School of Health, Mashhad University of Medical Sciences, Mashhad, Iran; 4 Department of Cardiology, Cardiovascular Diseases Research Centre, Birjand University of Medical Sciences, Birjand, Iran; 5 Department of Internal Medicine, School of Medicine, Birjand University of Medical Sciences, Birjand, Iran; Nathan S Kline Institute, UNITED STATES OF AMERICA

## Abstract

**Background:**

Sjogren’s syndrome (SS) is considered as a chronic, autoimmune disorder, that can present with various manifestations both intra and extra-glandular. Cognitive dysfunction is pivotal in recognizing neurological complications in SS.

**Methods:**

A study involving 44 SS cases and 37 controls was conducted to evaluate cognitive dysfunction further. Participants underwent multiple cognitive tests and blood tests for evaluation. Also, the β-site amyloid precursor protein cleaving enzyme 1 (BACE1), Interleukin 6 (IL-6), total antioxidant capacity (TAC), nitric oxide (NO), and malondialdehyde (MDA) serum levels were measured. Multiple analyses were done by PRISM 10 and SPSS 22.

**Results:**

The MoCA and SDLT scores were lower in Sjogren patients (P < 0.001). Serum BACE1, IL-6, NO, TAC, and MDA did not statistically vary in the SS patients. The only variables varied by medication therapy with methotrexate (MTX), hydroxychloroquine (HCQ), and prednisone were WBC count (P = 0.03) and triglyceride levels (in MTX and HCQ, P = 0.04), with no effect on neurocognitive factors. IL-6 was strongly correlated with the duration of symptoms (r = 0.99, P-value < 0.001). BACE1 had a positive correlation with IL-6 level (r = 0.4, P-value = 0.027).

**Conclusion:**

SS patients demonstrated significantly lower performance in neurocognitive tests, while BACE1 and inflammatory markers were not altered. This indicates that cognitive decline in SS is present but the mechanism still requires further evaluation. MTX, HCQ, and Prednisone use did not alter neurocognitive factors. Important correlations were found between hematological and cognitive tests in this study which provides new insights in the field of SS.

## 1. Introduction

Sjogren’s syndrome (SS) is characterized as a chronic, autoimmune mononuclear infiltration of exocrine glands. From a clinical perspective, more than half of the cases are diagnosed with other connective tissue diseases, and the symptoms are xerostomia and xerophthalmia (sicca syndrome) [1,2]. Over 33% of cases present with extra glandular involvement, with neurological manifestations being the most frequent [3,4]. While the neurological involvement in SS ranges from 8.5% to 70%, peripheral nervous system (PNS) involvement has been extensively studied in the matter of pathology, disease mechanism, and epidemiology compared to central nervous system (CNS) involvement [5,6]. These CNS involvements vary from aseptic meningitis, nonvasculitic autoimmune inflammatory meningoencephalitis (NAIM), cerebral vasculitis, encephalitis, and transverse myelitis [7,8].

As the subject of CNS manifestation in autoimmune disease and its potential mechanisms are not studied adequately, there exists a significant gap in our knowledge of this subject. Despite frequent evidence of CNS involvement in SS, a strong theory regarding the mechanism by which SS cases may develop cognitive dysfunction has yet to be established [6]. Despite these facts, there are multiple reports of mild cognitive difficulties often described as “brain fog” in SS patients which constitutes a crucial key point for investigation [5].

Despite the increasing prevalence of Alzheimer’s disease (AD) worldwide and several proposed mechanisms of pathology, no approved treatment is available for disease treatment or control [9]. Therefore, it is crucial to identify the risk factors and diseases associated with AD to better anticipate and mitigate future outbreaks. While tau formation and excessive production of Aβ accumulation are central to Alzheimer’s disease (AD) mechanisms, the SS mechanism of possible cognitive dysfunction remains under debate [10]. β-site amyloid precursor protein cleaving enzyme 1 (BACE1) is the predominant theory in AD pathogenesis, wherein this enzyme generates Aβ, and currently, multiple clinical trials are targeting BACE1 for controlling AD progression [11]. Although studies indicate that numerous clinical trials have been canceled due to safety challenges and ineffective cognitive improvement, this has led scientists to evaluate the BACE1 involvement in vascular, metabolic, and immune diseases to identify new treatment targets [12]. Based on this new trend following failures in BACE1 therapies, studies have expanded to further evaluate BACE1 in other diseases that reportedly cause cognitive dysfunction. Autoimmune diseases have been underrepresented in this discussion, with SS being one of the least considered in studies.

Since there is a lack of information on the molecular mechanism of SS and cognitive dysfunction, we aim to address this knowledge gap through this study. By designing a case-control study of SS patients, assessing their cognitive function using clinically approved tests, and collecting frequently monitored laboratory data, we intend to untie this knot by assessing the mechanism of BACE1 and Aβ formation pathway on cognitive impairment in SS individuals alongside inflammatory factors.

## 2. Materials and methods

### 2.1. Study population inclusion and exclusion criteria

In total, 81 participants were included in this study, of which 44 were Sjogren patients between 1^st^ April to 1^st^ September of 2024. The Sjogren patients were carefully selected from those referred to the rheumatologist’s clinic for routine disease follow-ups. A Written Informed consent to participate was obtained from all of the participants in the study. All participants received a written full description of the study’s process and requested data were confidentially encoded and collected. Any questions and doubts about the study were explained to the participants. The study involving human subjects, ethics approval, and consent to participate, was carried out by the Declaration of Helsinki and relevant guidelines in Birjand University of Medical Science. The Birjand University of Medical Science Ethics Committee approved all experimental protocols (IR.BUMS.REC.1402.410).

The inclusion criteria for Sjogren patients were a confirmed Sjogren Syndrome by a rheumatologist based on clinical signs and symptoms, as well as a positive (1:160 or above) fluorescent antinuclear antibody (FANA). Patients diagnosed with Sjogren Syndrome also had documented reports of positive anti-SSA and/or ‐SSB antibodies. The patients were also evaluated for a history of cognitive dysfunction diseases such as AD. All patients with a history of AD, head trauma, cardiovascular accident (CVD), or cerebrovascular accidents (CVA) were excluded. Patients referred to the clinic for routine disease follow-ups who met the above criteria were invited to voluntarily participate in the study.

For the control group, 37 participants were included. All participants were selected from cases that did not have any rheumatological diseases. Control cases were confirmed by a rheumatologist. The inclusion criteria for controls were: no prior history of any rheumatological diseases, and no history of AD, head trauma, CVD, or CVA. All control participants were required to have negative FANA and Anti‐SSA and ‐SSB antibodies. If a control met all the above criteria, they were invited to voluntarily participate in this study.

After obtaining informed consent, demographic data, current medication history, family history, signs, and symptoms were collected from the electronic system of the clinic where the data of all visits are documented. We also included the most recent blood test results of cases and controls. The laboratory tests were required to have been evaluated within the previous three months at the same laboratory institute for all participants. We included all cases and controls that were tested in the same laboratory to minimize variations in results. The laboratory data included; the most recent FANA for the case group, liver function tests (LFT), thyroid function tests (TFT), complete blood count (CBC), biochemistry tests; fasting blood glucose (FBG), calcium, vitamin D, creatinine, Urea, Lactate dehydrogenase (LDH), serology tests; rheumatoid factor (RF), C-reactive protein (CRP), one- and two-hour erythrocyte sedimentation rate (ESR), lipid profile and a urine test.

Furthermore, all participants were required to possess sufficient literacy skills to participate in tests that involved basic mathematical calculations and writing.

### 2.2. Interviewing terms and conditions

After meeting the criteria, patients and control participants were asked to complete A Mini-Mental State Examination (MMSE), Montreal Cognitive Assessment (MoCA), Serial Digit Learning Test (SDLT), and Pittsburgh Sleep Quality Index (PSQI). All patients were treated equally and the order of tests was the same. The test was collected in a quiet environment without stimulants. The same interviewer collected the data from all participants, and only the interviewer and the participant were present in the room. The interviewer was blind to participants’ status and was unaware of case or control designation. The evaluation time was between 4 and 8 PM daily for two months.

#### 2.2.1. Mini-Mental State Examination (MMSE).

Is a well-known screening instrument for the detection of cognitive impairment. The test comprises questions based on writing, basic mathematical calculations, drawing, and orientation to place and time. The MMSE has a maximum score of 30 and takes 5–10 minutes to answer. A score of 24 or above is considered normal, while a score of 23 or less indicates cognitive impairment [13]. The validated Persian version of the MMSE was used for the evaluation of participants [14]. All questions were asked at a consistent pace and tone for every participant without any confirmation or denial of responses.

#### 2.2.2. Montreal Cognitive Assessment (MoCA).

Designed by Dr Ziad Nasreddine in Montreal in 1995; was employed for the detection of mild cognitive impairment (MCI). The MoCA has been reported to be more sensitive for patients with mild symptoms and includes a clock drawing test, algorithms, reading, drawing, time and place orientation, and short-term memory evaluation [15]. With a maximum score of 30, a score of 26 or above is considered normal. A Persian-validated version of MoCA was administered to all participants [16]. The Interviewer performed the test that was previously described. For individuals with a diploma degree or lower, one point was added to the final score, as indicated by the MoCA guidelines.

#### 2.2.3. Serial Digit Learning Test (SDLT).

Is an effective test consisting of a 9-digit number sequence that is repeated for the participant 10 times. Each time a chance was given to the participant to recite the numbers in a correct order. The process is repeated 10 times. For enhanced differentiation and dispersion of cases, a modified version of SDLT was employed for evaluation. Participants got one point for a “nearly correct” answer, zero points for an incorrect answer, and two points for a completely right answer. In the modified version a “nearly correct” answer is defined as having only one digit omitted, added, or substituted, or a simple reversal of two adjacent digits [17]. The digits were read at a rate of one per second, and the answers were recorded.

**Pittsburgh Sleep Quality Index (PSQI)**: Is a widely recognized questionnaire for assessing sleep quality. For this study, a validated Persian version of the test was administered to the participants at the end of the session [18]. The questionnaire scores range from 0 to 21 divided by these subgroups: Sleep Latency (SL), Subjective Sleep Quality (SSQ), Habitual Sleep Efficiency (HSE), Use of Sleeping Medication (USM), Sleep Duration (SDu), Sleep Disturbances (SD), and Daytime Dysfunction (DD) [19]. To ensure accurate responses, the interviewer himself posed the questions and documented all responses.

### 2.3. Baseline examination

#### 2.3.1. Serum collection.

Following the interview, blood samples were obtained via venipuncture of an antecubital vein from cases and controls between 4–8:30 PM. The blood samples were collected in a 20mL vacuum tube and were sent for serum collection in an upright position following a standard protocol in five minutes. All samples were centrifuged at room temperature (20–25 °C), and three aliquots were extracted from the blood in 5mL Eppendorf tubes. After labeling the tubs, all samples were immediately frozen in a −87 °C freezer for subsequent analysis. All subsequent tests were conducted in a blinded manner. Samples were randomly tested and were measured based on assigned numbers.

#### 2.3.2. Enzyme-linked Immunosorbent Assay (ELISA) for Beta-secretase 1 (BACE1) Measurement.

The ELISA analysis of serum BACE1 was performed using biotin double sandwich technology by ZellBio (Germany) with a 96-well kit. After thawing the samples for 30 minutes at room temperature, sera were vortexed for approximately 30 seconds. For standard solutions, 60 µL of the standard solution was added to the well (96 pg/ml) and serially diluted to 48, 24,12,6, and 3 pg/ml by adding 60 µL standard diluent each time. A duplicate was included for each control sample by repeating the procedure. Subsequently, 50 µL of streptavidin-HRP were added to the wells. For the samples, 40 µL of the sample, 10 µL of BACE1 antibodies, and 50 µL of streptavidin-HRP were added to the wells, sealed, and incubated for 60 mins at 37 ºC. Following incubation, wells were washed 5 times according to the protocol with the diluted washing solution, and 100 µL of chromogen was added under limited light conditions to the wells. Samples were incubated for 20 minutes at 37 ºC. Subsequently, 50 µL of stop solution was added to all wells, and the absorbance of each well was measured at the wavelength of 450 nm. The sensitivity of the kit was reported 0.4 pg/ml, and the assay range was mentioned to be 0.3 pg/ml to 96 pg/ml.

#### 2.3.3. Enzyme-linked Immunosorbent Assay (ELISA) for Interleukin 6 (IL-6) Measurement.

The ELISA analysis of serum IL-6 was conducted using biotin double sandwich technology with a 96-well kit from Karmania pars gen (catalog number: KPG-HIL6). Thirty SS paints and 20 controls were randomly selected, and serum IL6 was evaluated due to time limitations and potential time variations. After thawing the samples for 30 minutes at room temperature, sera were vortexed for approximately 30 seconds. For standard solutions, 10 µL of the standard solution was vortexed with 1.5 cc of elution buffer. 50 µL of elution buffer was added to the wells A1 to D1, and 50 µL of prepared standard solution was added to D1 and E1, Subsequently, 50 µL was transferred from D1 to C1, 50 µL from C1 to B1 and finally, and 50 µL from B1 was discarded. 50 µL of serum samples were added to the wells. Then samples were placed on a shaker for 50 minutes at 100 rounds per minute (RPM) at room temperature. The plate was washed with washing solution three times, and 50 µL conjugated antibodies by American R&D were added to all wells except A1. The plate was incubated again on a 100 RPM shaker for 50 minutes at room temperature. Subsequently, the plate was washed 3 times with washing solution, and 50 µL of HRP-Avidin was added to all wells except A1, followed by incubation under the same condition for 30 minutes. Finally, after washing with washing solution 5 times, adding 50 µL of substrate, waiting for 10 minutes and adding 25 µL of stop solution the results were measured at the wavelength of 450 nm. The sensitivity of the kit was reported as 2 pg/ml.

#### 2.3.4. Malondialdehyde (MDA) measurement.

MDA assay kit from Karmania pars gen with a 96-well kit was utilized (catalog number: KPG-MDA). Raw materials were provided by Sigma and Merck Biotech Inc.

After thawing the samples for 30 minutes at room temperature, sera were vortexed for approximately 30 seconds. Solutions A, B, and C were mixed in a ratio of 1:1:2 to create the work solution. 20.5 µL of the standard solution was mixed with 1 cc of 96% ethanol and 49 cc of distilled water was added. The solution was then added to microtubes in dilutions of 50, 10, 5, 2.5, 1.25, 0.62, 0.31, and 0.15 micromoles. 200 µL of previously prepared work solution was added to all standards, blank, and samples. 100 µL serum samples, distilled water (for a negative control), and standard were added to each microtube. All microtubes were incubated at 90c for 60 minutes and then for 10 minutes at room temperature. Subsequently, they were centrifuged for 10 minutes at 1000 RPM, and finally, 150 µL from each microtube was added to wells and measured at a wavelength of 535 nm immediately.

#### 2.3.5. Total Antioxidant Capacity (TAC) measurement.

TAC assay kit from Karmania pars gen with a 96-well kit was utilized (catalog number: KPG-FRAP).

The TAC test was based on the Ferric ferric-reducing ability of Plasma (FRAP). After thawing the samples for 30 minutes at room temperature, sera were vortexed for approximately 30 seconds. The work solution was prepared according to the catalog instructions, and 10 µL of standards, samples and distilled water were added to the wells along with 140 µL of work solution. The plate was incubated at 37 C° for 5 minutes, and the samples were measured at a wavelength of 593 nm immediately.

#### 2.3.6. Nitric Oxide (NO) measurement.

NO assay kit from Karmania pars gen with a 96-well kit was utilized (catalog number: KPG-Nob).

After thawing the samples for 30 minutes at room temperature, sera were vortexed for approximately 30 seconds. The work solution was prepared according to the catalog instructions, and 100 µL of work solution was added to microtubes. 30 µL of samples and distilled water were added to the microtubes for samples and blank tests, respectively. For standard solutions, 5 standard solutions (100 µmol, 50 µmol, 25 µmol, 12.5 µmol, and 6.25 µmol) were prepared according to the instructions. The plate was incubated and shaken for 10 minutes in a dark environment, and subsequently, 150 µL of solution was added to the microtubes. 150 µL of the final solution was added to the wells and the samples were measured at a wavelength of 450nm immediately.

### 2.4. Statistical analysis

Characteristics of the study population were described using means, standard deviation (SD), and numbers (%) for categorical variables. A comparison of baseline characteristics between the case and control groups employed the student’s t-test for continuous variables, the chi-square test for categorical variables, and the Mann-Whitney test for skewed variables. Violin plots for key variables were also provided. Multiple linear regression was performed for variables and some were reported in the result section. Multiple variables were considered for adjustment. Cognitive function assessments (MMSE, MoCA, SDLT) were adjusted based on age, education, and the administration of MTX, Prednisone, and HCQ. Vitamin D levels were adjusted based on the intake of Vitamin D supplements, MTX, Prednisone, and HCQ. TSH levels were adjusted for the administration of Levothyroxine, MTX, Prednisone, and HCQ. Sleep quality scores were adjusted considering the administration of Benzodiazepine, Psychiatric Drugs, MTX, Prednisone, and HCQ. For HDL, LDL, TG, and TC levels, adjustments were made based on the administration of Atorvastatin, MTX, Prednisone, and HCQ. Calcium levels were adjusted for any type of calcium supplement intake, as well as MTX, Prednisone, and HCQ administration. Lastly, ESR1, ESR2, CRP, and RF levels were adjusted for the administration of MTX, Prednisone, and HCQ. Analysis was conducted by SPSS Version 22. Pearson and Spearman correlation coefficients were utilized to examine the relationships between evaluated factors. For the correlogram, Spearman correlation coefficients were utilized as some factors such as MMSE, SDLT, and Moca did not follow the Gaussian distribution. For scatter plots, Pearson correlation coefficients were utilized. Visualization of plots was performed by PRISM 10. A P-value less than 0.05 was considered statistically significant in this study.

## 3. Results

### 3.1. Study population: demographics and baseline variables

A total of 81 participants were included in this study, with 44 in the Sjogren patient group and 37 in the control group (Table 1). In most cases Sjogren is female, and all the subjects in both patient and healthy control groups were selected as female. The mean age of Sjogren was 49.66 ± 10.93 years, and the control group was 48.19 ± 9.75 years with no statistically significant age-related differences between the patients and control subjects (P-value = 0.84). The average BMI in the Sjogren patient group was 25.61 ± 4.36 *kg/m*^*2*^, which did not differ significantly from the control group, where the mean BMI was 27.50 ± 5.05 *kg/m*^*2*^ (P-value = 0.07). The number of siblings reported by participants was 4.89 ± 2.82 in the Sjogren patient group and 4.70 ± 2.25 in the control group (P-value = 0.87), indicating no significant difference between the two groups.

**Table 1:** Demographic characteristics of the sjögren syndrome patients and controls.

Characteristics	Total	Patients (n = 44)	Control (n = 37)	p-value
General				
Gender: Female/Male *(number)*	81/0	44/0	37/0	
Age *(years ± SD)*	49.44 ± 10.35	49.66 ± 10.93	48.19 ± 9.75	0.84^*^
Educational level				0.605^***^
Below Associate *(number, %)*	18 (22%)	10 (23%)	8 (22%)	
Associate/Diploma *(number, %)*	23 (28%)	14 (32%)	9 (24%)	
Bachelor’s Degree *(number, %)*	29 (36%)	13 (29%)	16 (43%)	
Master, or Higher Degree *(number, %)*	11 (14%)	7 (16%)	4 (11%)	
Siblings *(numbers ± SD)*	4.80 ± 2.56	4.89 ± 2.82	4.70 ± 2.25	0.87^**^
Weight *(kg ± SD)*	67.83 ± 11.15	65.93 ± 11.30	70.14 ± 10.67	0.09^*^
Height *(cm ± SD)*	160.39 ± 6.35	160.55 ± 6.71	160.19 ± 5.97	0.80^*^
BMI *(kg/*$m^{2}\;\pm \;SD)$	26.46 ± 1.59	25.61 ± 4.36	27.50 ± 5.05	0.07^*^
Sjogren’s Disease Characteristics				
Duration of Symptoms *(years ± SD)*	**_**	8.74 ± 6.36	**_**	**_**
Duration of Diagnosis *(years ± SD)*	**_**	6.74 ± 5.05	**_**	**_**
Duration of Treatment *(years ± SD)*	**_**	6.80 ± 4.83	**_**	**_**
Dental Visits *(per year ± SD)*	2.06 ± 2.10	3.01 ± 2.18	0.93 ± 1.33	<0.001^**^
Family History of Autoimmune Disorders *(number, %)*	26 (32%)	16 (36%)	10 (27%)	0.37^***^
Family History of Neurologic Disorders *(number, %)*	19 (23%)	11 (25%)	8 (22%)	0.72^***^
History of Neurology Disorder *(number, %)*	2 (0.02%)	2 (0.04%)	0 (0%)	0.2^***^
Other Autoimmune Diseases *(number, %)*	22 (27%)	22 (50%)	0 (0%)	< 0.001^***^
Recent Medication History				
Prednisolone Recipients	30 (37%)	29 (66%)	1(0.03%)	< 0.001^***^
MTX Recipients	18 (22%)	18 (41%)	0 (0%)	< 0.001^***^
HCQ Recipients	27 (33%)	27 (61%)	0 (0%)	< 0.001^***^
Azathioprine Recipients	2 (0.02%)	2 (0.04%)	0 (0%)	0.19^***^
Psychiatric medicines Recipients	17 (21%)	6 (14%)	11 (30%)	0.056^***^
Levothyroxine Recipients	13 (16%)	7 (16%)	6 (16%)	0.97^***^
Benzodiazepine Recipients	9 (11%)	7 (16%)	2 (0.05%)	0.17^***^
Vitamin D Supplements	18 (22%)	9 (20%)	9 (24%)	0.68^***^
Omega 3 Supplements	14 (17%)	9 (20%)	5 (13%)	0.29^***^
Calcium Supplements	29 (36%)	22 (50%)	7 (19%)	0.004^***^

*Independent sample t test, ^**^Mann-Whitney, ^***^Chi-square

SD: Standard Deviation, BMI: Body Mass Index, MTX: Methotrexate, HCQ: Hydroxychloroquine

A statistically significant difference was observed in the frequency of dental visits between the two groups. On average, the Sjogren patient group visited the dentist 3.01 ± 2.18 times in the last year, which was significantly higher than that of the control group, whose mean visit numbers were 0.93 ± 1.33 (P-value < 0.001). Also, Sjogren patients demonstrated a mean duration of disease-related symptoms of 8.74 ± 6.36 years, average years of diagnosis of 6.74 ± 5.05, and the mean duration of treatment of 6.80 ± 4.83 years.

The chi-square test for categorical variables showed that family history of autoimmune disease and neurologic disorders among first-degree relatives were not statistically significant (P-Value = 0.37 and 0.72 respectively). Other autoimmune disorders were significantly more prevalent in the Sjogren’s syndrome group, as SS frequently co-occurs with rheumatoid arthritis (P-value <0.001). The utilization of prescribed Vitamin D and Omega 3 supplements did not differ statistically between the two groups, whereas Calcium supplements were more frequently prescribed based on abnormal bone density scans (P-value = 0.004). Prescription of MTX, HCQ, and Prednisolone was statistically higher in the Sjogren’s syndrome group (P-value <0.001), in contrast with Azathioprine, Levothyroxine, Benzodiazepines, and psychiatric medications. These findings suggest that diagnosed psychiatric health issues requiring treatment are not altered in SS.

### 3.2. Routine blood tests (hematological, metabolic, electrolytes, minerals, and vitamins)

In routine blood tests for the past six months (Table 2), WBC levels were significantly lower in Sjogren patients (6.40 ± 1.57 $10^{3}$*/µL* vs. 7.16 ± 1.54 $10^{3}$*/µL*, P-value = 0.03). Additionally, monocyte percentages were notably higher in the Sjogren group compared to the control group (8.88 ± 9.23 $10^{3}$*/µL* vs. 6.56 ± 1.47 $10^{3}$*/µL*, P-value = 0.04), suggesting variations in hematological profiles associated with Sjogren’s Syndrome. Conversely, there were no statistically significant differences in the RBC count and its related parameters, including Hgb, HCT, MCV, MCH, and MCHC, between the two groups.

**Table 2:** Clinical and laboratory characteristics of the sjögren syndrome patients and controls.

	Total *(mean ± SD)*	Patients *(mean ± SD)*	Control *(mean ± SD)*	p-value
Metabolic Panel				
FBS *(mg/dL)*	88.88 ± 14.02	88.17 ± 14.73	89.72 ± 13.31	0.38^**^
Urea *(mg/dL)*	28.37 ± 8.87	27.22 ± 9.13	29.51 ± 8.59	0.09^**^
BUN *(mg/dL)*	13.25 ± 4.23	12.78 ± 4.42	13.66 ± 4.07	0.19^**^
Cr *(mg/dL)*	0.07 ± 0.26	0.12 ± 0.33	0.03 ± 0.16	0.12^**^
Lipid Profile				
Total Cholesterol *(mg/dL)*	183.35 ± 43.96	173.78 ± 33.38	194.36 ± 52.01	0.09^**^
Triglycerides *(mg/dL)*	135.77 ± 79.01	121.39 ± 65.88	152.33 ± 90.05	0.12^**^
HDL Cholesterol *(mg/dL)*	48.42 ± 10.14	49.00 ± 8.69	47.70 ± 11.78	0.24^**^
LDL Cholesterol *(mg/dL)*	103.68 ± 32.02	95.08 ± 25.33	114.22 ± 36.39	0.01^*^
Liver Function Tests				
AST *(IU/L)*	20.41 ± 8.55	21.66 ± 10.14	18.94 ± 6.02	0.12^**^
ALT *(IU/L)*	21.31 ± 10.79	21.34 ± 11.32	21.27 ± 10.28	0.88^**^
ALP *(U/L)*	94.95 ± 42.85	89.75 ± 42.70	102.95 ± 42.70	0.15^**^
Thyroid Function Tests				
TSH *(mIU/L)*	2.02 ± 1.41	2.22 ± 1.39	1.82 ± 1.42	0.17^**^
T3 *(µg/dL)*	114.22 ± 27.79	113.52 ± 31.55	115.77 ± 18.32	0.65^**^
T4 *(µg/dL)*	6.09 ± 2.71	2.95 ± 0.34	7.86 ± 1.57	<0.001^**^
CBC with WBC Differential				
WBC ($10^{3}$*/µL)*	6.75 ± 1.59	6.40 ± 1.57	7.16 ± 1.54	0.03^*^
RBC ($10^{6}$*/µL)*	4.07 ± 0.53	4.00 ± 0.58	4.17 ± 0.45	0.17^**^
Hgb *(g/dL)*	13.11 ± 1.87	12.88 ± 1.32	13.40 ± 0.94	0.06^**^
Hct *(%)*	39.88 ± 3.30	39.28 ± 3.67	40.60 ± 2.67	0.08^*^
MCV *(fL)*	87.72 ± 5.87	88.50 ± 6.57	86.79 ± 4.83	0.11^**^
MCH *(pg)*	29.02 ± 2.13	29.21 ± 2.49	2.80 ± 1.62	0.13^**^
MCHC *(g/dL)*	33.10 ± 1.10	33.02 ± 1.17	33.02 ± 1.02	0.59^**^
PLT ($10^{3}$*/µL)*	279.76 ± 75.97	268.11 ± 62.69	293.73 ± 88.29	0.08^**^
RDW-CV *(%)*	13.01 ± 1.10	13.00 ± 1.02	13.02 ± 1.21	0.88^**^
Neutrophils *%*	54.74 ± 9.58	54.22 ± 11.08	55.34 ± 7.62	0.61^*^
Lymphocytes *%*	34.64 ± 8.27	34.40 ± 8.72	34.91 ± 7.84	0.79^*^
Monocytes *%*	7.80 ± 6.87	8.88 ± 9.23	6.56 ± 1.47	0.04^**^
Eosinophils *%*	2.50 ± 3.46	2.87 ± 4.26	2.09 ± 2.19	0.90^**^
Basophils *%*	0.11 ± 0.84	0.18 ± 1.13	0.03 ± 0.17	0.93^**^
Electrolytes, Minerals, and Vitamins				
Ca *(mg/dL)*	9.59 ± 0.39	9.46 ± 0.38	9.77 ± 0.32	0.001^**^
P *(mg/dL)*	3.52 ± 0.69	3.49 ± 0.65	3.58 ± 0.77	0.65^**^
Vitamin D *(ng/mL)*	34.84 ± 12.21	33.29 ± 11.78	36.48 ± 12.61	0.22^**^

*independent sample t test,

**Mann-Whitney, SD: Standard Deviation, CBC: Complete Blood Count, WBC: White Blood Cell, RBC: Red Blood Cell, BUN: Blood Urea Nitrogen, ALP: Alkaline Phosphatase, ALT: Alanine Aminotransferase, AST: Aspartate Aminotransferase, TSH: Thyroid Stimulating Hormone, FBS: Fasting Blood Sugar, T3: Triiodothyronine, T4: Thyroxine, Ca: Calcium, P: Phosphorus, HDL: High-Density Lipoprotein, LDL: Low-Density Lipoprotein, PLT: Platelet Count, RDW-CV: Red Cell Distribution Width–Coefficient of Variation, Hgb: Hemoglobulin, Hct: Hematocrit, MCV: Mean Corpuscular Volume, MCH: Mean Corpuscular Hemoglobin, MCHC: Mean Corpuscular Hemoglobin Concentration, Cr: Creatinine

Furthermore, no statistically significant differences were observed in the metabolic parameters (FBS, Urea, Creatinine, BUN) between the Sjogren patients and the control group. In the lipid profile, LDL was significantly lower in Sjogren’s compared to healthy controls (95.08 ± 25.33 *mg/dL* vs. 114.22 ± 36.39 *mg/dL*, P-value = 0.01). In thyroid function tests, T4 levels were significantly lower in Sjogren patients compared to the control group (2.95 ± 0.34 *µg/dL* vs. 7.86 ± 1.57 *µg/dL*, P-value < 0.001).

Lastly, Calcium levels were significantly lower in Sjogren’s compared to the controls (9.46 ± 0.38 *mg/dL* vs. 9.77 ± 0.32 *mg/dL*, P-value = 0.001). However, no significant differences were observed in vitamin D levels between the two groups.

### 3.3 Neurocognitive assessments

Neurocognitive assessments include the MMSE, MoCA, Sleep Quality Assessment, and SDLT tests. The mean MoCA score in the control group was 27.30 ± 2.72, whereas in Sjogren patients, the mean score was significantly lower at 24.14 ± 4.34 (P-value < 0.001). SDLT scores were significantly lower in the Sjogren’s group, compared to the control group (5.34 ± 6.55 vs 10.54 ± 5.54, P-value < 0.001). The mean MMSE score was 26.77 ± 4.19 for the Sjogren patients and 28.35 ± 1.82 in controls with no statistical difference (P-value = 0.10). Regarding sleep quality, Sjogren’s syndrome patients reported a mean score of 8.20 ± 3.48, compared to 7.03 ± 3.67 for the control group with no significant differences (P-value = 0.14). Data from neurocognitive assessments are shown in Table 3.

**Table 3:** Neurocognitive assessments of the sjögren syndrome patients and controls.

	Total *(mean ± SD)*	Patients (n = 44) *(mean ± SD)*	Control (n = 37) *(mean ± SD)*	p-value
MMSE	27.49 ± 3.40	26.77 ± 4.19	28.35 ± 1.82	0.10^**^
Sleep	7.67 ± 3.60	8.20 ± 3.48	7.03 ± 3.67	0.14^*^
MoCA	25.58 ± 4.00	24.14 ± 4.34	27.30 ± 2.72	<0.001^**^
SDLT	7.72 ± 6.61	5.34 ± 6.55	10.54 ± 5.54	<0.001^**^

*Independent sample t test, ^**^Mann-Whitney

SD: Standard Deviation, MMSE: Mini-Mental State Examination, MoCA: Montreal Cognitive Assessment, SDLT: Serial Digit Learning Test

### 3.4. Inflammatory biomarkers and BACE 1

**Fig 1:**
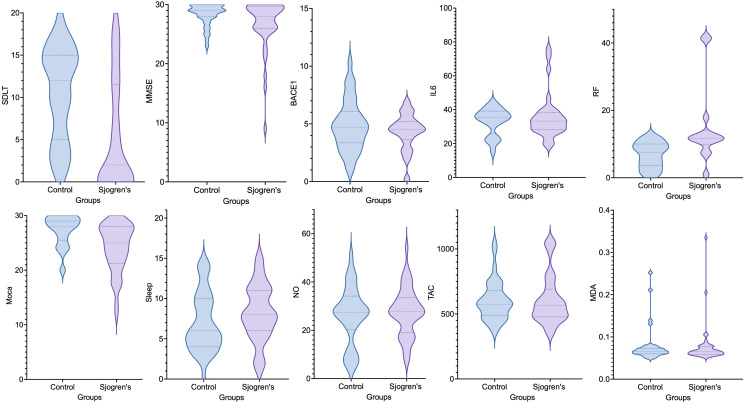
Violin Plots for Cognitive Assessments, Inflammatory, and Oxidative Stress Markers in Sjogrem Syndrom Patients and Controls. BACE1 (pg/mL), IL-6 (pg/mL), MDA (µmol/L), NO (*µmol/L),* RF *(titer),* TAC µmol TE/L. MMSE: Mini-Mental State Examination, MoCA: Montreal Cognitive Assessment, Sleep: Pittsburgh Sleep Quality Index, SDLT: Serial Digit Learning Test, BACE1: Beta-Site Amyloid Precursor Protein Cleaving Enzyme1, IL-6: Interleukin-6, MDA: Malondialdehyde, NO: Nitric Oxide, TAC: Total Antioxidant Capacity, RF: Rheumatoid factor.

Table 4 presents the comparison of oxidative stress and inflammatory biomarkers between Sjogren patients and the control group. In the matter of BACE1 as a marker in amyloid-ß formation, the serum level differences observed between Sjogren’s patients and the control group were not statistically significant (P-value = 0.22). Serum Interleukin 6 levels were not statistically different between the two groups (P-value = 0.68) as well as other inflammatory markers such as MDA (P-value = 0.86), TAC (P-value = 0.79), NO (P-value = 0.55), ESR (P-value = 0.62) and CRP (P-value = 0.92). The similarity between the two groups in non-specific inflammatory markers was predictable, as control patients were selected from individuals referring to the rheumatology clinic for evaluation. As expected, RF was significantly elevated in Sjogren patients compared to controls (14.62 ± 11.70 vs. 6.61 ± 3.61, P-value = 0.001). Fig 1 shows the distribution of inflammatory biomarkers, BACE1, and neurocognitive assessments by violin plots. **Violin Plots for Cognitive Assessments, Inflammatory, and Oxidative Stress Markers in Sjogrem Syndrom Patients and Controls.**

**Table 4:** Oxidative stress and inflammatory markers of the sjögren syndrome patients and controls.

	Total *(mean ± SD)*	Patients (n = 44) *(mean ± SD)*	Control (n = 37) *(mean ± SD)*	p-value
ESR/1h *(mm/1h)*	13.93 ± 10.20	13.51 ± 10.39	14.37 ± 10.19	0.62^**^
ESR/2h *(mm/2h)*	26.14 ± 15.75	23.51 ± 15.48	28.54 ± 15.85	0.15^**^
RF *(titer)*	9.56 ± 8.46	14.62 ± 11.70	6.61 ± 3.61	0.001^**^
CRP *(mg/L)*	5.33 ± 5.12	5.00 ± 4.47	5.69 ± 5.79	0.92^**^
BACE1 *(pg/mL)*	4.51 ± 1.86	4.27 ± 1.55	4.80 ± 2.17	0.22^*^
IL-6 *(pg/mL)*	34.66 ± 11.49	35.72 ± 13.39	33.08 ± 7.91	0.68^**^
MDA *(nmol/L)*	77.34 ± 46.91	78.30 ± 42.05	0.08 ± 0.04	0.86^**^
NO (*µmol/L)*	27.06 ± 10.88	27.73 ± 9.97	26.26 ± 11.97	0.55^*^
TAC µmol TE/L	607.16 ± 17.3.78	610.90 ± 190.72	602.68 ± 153.67	0.79^**^

*Independent sample t test, ^**^Mann-Whitney

SD: Standard Deviation, BACE1: Beta-Site Amyloid Precursor Protein Cleaving Enzyme1, IL-6: Interleukin-6, MDA: Malondialdehyde, NO: Nitric Oxide, TAC: Total Antioxidant Capacity, RF: Rheumatoid Factor, CRP: C-Reactive Protein, ESR: Erythrocyte Sedimentation Rate

### 3.5. Multiple linear regression analysis

As indicated in Table 5, multiple linear regression analysis revealed the strongest association with SDLT scores, which were significantly lower in the SS patients’ group (B = −7.04, SE = 1.65, Beta = −0.54, P-value < 0.001 respectively). Similarly, MoCA scores were significantly lower in the Sjogren patients’ group (B = −3.64, SE = 1.00, Beta = −0.46, P-value = 0.001 respectively), indicating that SS is a strong predictor of cognitive deficit. MoCA scores were 3.64% lower in the Sjogren group compared to controls, while SDLT scores decreased by 7.04% in the Sjogren group, which was nearly double the decrease observed in the Moca scores. Other variables, such as MMSE did not show significant findings. RF levels were significantly higher in the SS patients’ group (B = 0.02, SE = 0.007, Beta = 0.31, P-value = 0.01). Other non-specific inflammatory biomarkers, including CRP, MDA, TAC, ESR, IL-6, and NO, showed no significant associations with group status (P-value > 0.05). Cr levels approached significance, with higher levels observed in the Sjogren’s group (B = 0.28, SE = 0.14, Beta = 0.15, P-value = 0.06). However, calcium (P-value = 0.29), and other metabolic markers showed no statistically significant difference.

**Table 5:** Multiple linear regression analysis of cognitive assessments, inflammatory and oxidative stress markers, and clinical tests adjusted for covariates in the sjögren syndrome patients.

Dependent	Independent	B (SE)	Beta	p-value
Neurocognitive Assessments				
MMSE	group	−1.2(0.92)	−0.17	0.20
Sleep	group	−0.003(0.01)	−0.02	0.78
MoCA	group	−3.64(1.00)	−0.46	0.001^***^
SDLT	group	−7.04(1.65)	−0.54	<0.001^***^
Oxidative Stress and Inflammatory Markers				
BACE1	group	−0.007(0.02)	−0.03	0.75
IL-6	group	0.004(0.004)	0.09	0.38
MDA	group	−0.41(0.90)	−0.04	0.64
TAC	group	0.002(0.0002)	0.07	0.37
NO	group	0.003(0.004)	0.08	0.33
ESR	group	−0.003(0.004)	−0.06	0.49
RF	group	0.02(0.007)	0.31	0.01
CRP	group	−0.006(0.008)	−0.06	0.47
BMI	group	−0.01(0.01)	−0.09	0.28
WBC	group	−0.02(0.03)	−0.05	0.52
Neutrophil %	group	−0.002(0.004)	−0.04	0.59
Lymphocyte %	group	−0.0002(0.005)	−0.003	0.97
Creatinine	group	0.28(0.14)	0.15	0.06
Total Cholesterol	group	−0.0002(0.001)	−0.02	0.77
TSH	group	0.04(0.03)	0.10	0.16
Calcium	group	−0.12(0.11)	−0.10	0.29
Vitamin D	group	0.001(0.003)	0.003	0.97

SE: Standard Error, MMSE: Mini-Mental State Examination, MoCA: Montreal Cognitive Assessment, SDLT: Serial Digit Learning Test, BACE1: Beta-Site Amyloid Precursor Protein Cleaving Enzyme1, IL-6: Interleukin-6, MDA: Malondialdehyde, NO: Nitric Oxide, TAC: Total Antioxidant Capacity, RF: Rheumatoid Factor, CRP: C-Reactive Protein, ESR: Erythrocyte Sedimentation Rate, BMI: Body Mass Index, WBC: White Blood Cell, TSH: Thyroid Stimulating Hormone

Covariates Adjustments include the following: Cognitive assessments; MMSE, MoCA, SDLT: Adjusted for age, education, medication use (e.g., MTX, Prednisolone, and HCQ); Sleep quality: Adjusted for medication use (e.g., Benzodiazepine, Psychiatric Drugs, MTX, Prednisolone, and HCQ). Inflammatory Markers (ESR, CRP, RF): Adjusted for medication use (e.g., MTX, Prednisolone, and HCQ). Clinical Tests; Total Cholesterol: Adjusted for medication use (e.g., Atorvastatin, MTX, Prednisolone, and HCQ); TSH: Adjusted for medication use (e.g., Levothyroxine, MTX, Prednisolone, and HCQ); Calcium: Adjusted for medication use (e.g., any type of Calcium supplements, MTX, Prednisolone, and HCQ); Vitamin D: Adjusted for medication use (e.g., any type of vitamin D supplements, MTX, Prednisolone, and HCQ)

### 3.6. Disease-Modifying Anti-Rheumatic Drugs (DMARDs) and prednisolone effects

The analysis of cognitive and biochemical markers among Sjogren patients was stratified based on the recipients of DMARDs (MTX and HCQ) and prednisolone. Comparisons between recipients and non-recipients revealed that WBC counts were consistently lower in medicine recipients across all groups (Prednisolone, MTX, and HCQ) compared to non-recipients (P-value = 0.03 for all three). Additionally, only in DMARDs, MTX (101.75 ± 57.71 vs. 135.68 ± 69.01, P-value = 0.04) and HCQ recipients (110.44 ± 61.98 vs. 142.46 ± 70.52, P-value = 0.04) had significantly lower triglyceride levels. However, no significant differences were observed in cognitive markers, including MMSE, MoCA, and SDLT, between DMARDs and Prednisolone recipients and non-recipients. Table 6 illustrates the data on the effect of medication therapy in evaluated markers.

**Table 6:** Disease-Modifying Anti-Rheumatic Drugs (DMARDs) and prednisolone effects.

	Total (n = 44) *(mean ± SD)*	Prednisolone Recipients (n = 29) *(mean ± SD)*	Non-Prednisolone Recipients (n = 15) *(mean ± SD)*	p-value	MTX Recipients (n = 18) *(mean ± SD)*	Non- MTX Recipients (n = 26) *(mean ± SD)*	p-value	HCQ Recipients (n = 27) *(mean ± SD)*	Non-HCQ Recipients (n = 17) *(mean ± SD)*	p-value
Neurocognitive Assessments										
MMSE	26.77 ± 4.19	26.86 ± 4.9	26.6 ± 2.41	0.58^**^	28.17 ± 2.33	25.81 ± 4.92	0.58^**^	26.52 ± 5.01	27.18 ± 2.48	0.58^**^
Sleep	8.2 ± 3.49	8.48 ± 3.84	7.67 ± 2.71	0.27^*^	8.39 ± 3.18	8.08 ± 3.74	0.27^*^	8.67 ± 3.57	7.47 ± 3.32	0.27^*^
MoCA	24.14 ± 4.34	24.79 ± 4.69	22.87 ± 3.35	0.58^**^	25.67 ± 3.66	23.08 ± 4.52	0.57^**^	24.15 ± 4.87	24.12 ± 3.48	0.57^**^
SDLT	5.34 ± 6.56	6.38 ± 6.77	3.33 ± 5.79	0.51^**^	9.06 ± 7.64	2.77 ± 4.17	0.51^**^	5.93 ± 6.9	4.41 ± 6.05	0.51^**^
Oxidative Stress and Inflammatory Markers										
BACE1 *(pg/mL)*	4.27 ± 1.56	4.29 ± 1.57	4.23 ± 1.58	0.16^*^	4.24 ± 1.21	4.3 ± 1.78	0.16^*^	4.01 ± 1.46	4.7 ± 1.66	0.16
IL-6 *(pg/mL)*	35.72 ± 13.39	36.47 ± 12.75	33.96 ± 15.44	0.44^**^	35.32 ± 15.84	35.92 ± 12.44	0.44^**^	32.33 ± 7.13	40.81 ± 18.63	0.44^**^
MDA *(nmol/L)*	77.35 ± 46.92	81.9643 ± 57.08	68.73 ± 13.98	0.80^**^	69.83 ± 12.41	82.76 ± 60.58	0.80^**^	80.3 ± 57.97	72.38 ± 17.19	0.80^**^
TAC	610.91 ± 190.73	606.83 ± 193.46	618.51 ± 191.97	0.44^**^	583.7 ± 154.85	630.49 ± 213.82	0.44^**^	584.87 ± 170.77	654.84 ± 219.16	0.44^**^
NO	27.73 ± 9.98	27.43 ± 9.99	28.3 ± 10.26	0.23^*^	27.82 ± 8.13	27.67 ± 11.28	0.23^*^	26.31 ± 9.67	30.13 ± 10.33	0.23^*^
RF *(titer)*	14.63 ± 11.71	13.56 ± 11.15	16.56 ± 13.75	0.36^**^	15.4 ± 15.45	14.2 ± 10.14	0.85^*^	14 ± 12.85	15.26 ± 11.45	0.36^**^
CRP *(mg/L)*	5.00 ± 4.47	5.41 ± 4.94	4.16 ± 3.32	0.92^**^	5.85 ± 5.77	4.36 ± 3.17	0.86^*^	5.1 ± 5.24	4.84 ± 2.98	0.86^*^
ESR/1h *(mm/1h)*	13.52 ± 10.33	14.8 ± 11.78	11.15 ± 6.69	0.71^**^	15.64 ± 12.63	12.22 ± 8.7	0.71^**^	13.1 ± 9.35	14.07 ± 11.78	0.78^*^
ESR/2h *(mm/2h)*	23.51 ± 15.48	24.39 ± 17.16	22 ± 12.68	0.29^**^	25.5 ± 18.36	22.19 ± 13.64	0.29^**^	25.28 ± 16.07	20.87 ± 14.84	0.45^*^
WBC ($10^{3}$*/µL)*	6.41 ± 1.57	6.35 ± 1.55	6.53 ± 1.66	0.03^*^	6.35 ± 1.37	6.45 ± 1.73	0.03^*^	6 ± 1.56	7.07 ± 1.4	0.03^*^
RBC ($10^{6}$*/µL)*	4 ± 0.58	3.89 ± 0.62	4.21 ± 0.42	0.28^**^	3.88 ± 0.49	4.08 ± 0.64	0.27^**^	3.92 ± 0.56	4.13 ± 0.62	0.27^**^
PLT ($10^{3}$*/µL)*	268.12 ± 62.69	271.46 ± 54.78	261.43 ± 78.02	0.77^*^	275.71 ± 47.25	262.96 ± 71.81	0.77^*^	270.35 ± 50.31	264.5 ± 80.63	0.77^*^
FBS *(mg/dL)*	88.18 ± 14.74	90.19 ± 15.32	84.15 ± 13.12	0.47^**^	83.07 ± 11.09	91.38 ± 16	0.48^**^	86.12 ± 12.52	91.86 ± 17.97	0.24^*^
Urea *(mg/dL)*	27.23 ± 9.13	26.84 ± 10.09	28.2 ± 6.46	0.20^**^	24.29 ± 4.32	29.19 ± 10.93	0.22^**^	25.55 ± 7.26	30.08 ± 11.41	0.15^*^
BUN *(mg/dL)*	12.79 ± 4.43	12.54 ± 5.13	13.33 ± 2.34	0.40^**^	10.61 ± 1.92	14.12 ± 5.02	0.40^**^	12.09 ± 3.4	13.78 ± 5.59	0.32^*^
Cr *(mg/dL)*	0.12 ± 0.33	0.11 ± 0.31	0.15 ± 0.37	0.57^**^	0.06 ± 0.24	0.17 ± 0.38	0.31^**^	0.08 ± 0.28	0.19 ± 0.4	0.32^*^
Total Cholesterol *(mg/dL)*	173.79 ± 33.39	167.56 ± 33.39	189.09 ± 29.37	0.39^*^	171.25 ± 33.61	175.64 ± 33.89	0.39^*^	170.4 ± 36.41	180.31 ± 26.75	0.40^*^
Triglycerides *(mg/dL)*	121.39 ± 65.89	116.93 ± 57.7	132.36 ± 84.96	0.16^*^	101.75 ± 57.71	135.68 ± 69.01	0.04^**^	110.44 ± 61.98	142.46 ± 70.52	0.04^**^
HDL Cholesterol *(mg/dL)*	49 ± 8.7	49.85 ± 9.01	46.91 ± 7.88	0.72^*^	47.13 ± 7.68	50.36 ± 9.3	0.73^*^	48.64 ± 8.59	49.69 ± 9.21	0.73^*^
LDL Cholesterol *(mg/dL)*	95.09 ± 25.34	88.35 ± 22.75	111.64 ± 24.59	0.45^*^	96.21 ± 29.56	94.27 ± 22.48	0.45^*^	92.86 ± 26.04	99.38 ± 24.34	0.46^*^
Ca *(mg/dL)*	9.47 ± 0.39	9.41 ± 0.39	9.6 ± 0.35	0.25^*^	9.46 ± 0.42	9.47 ± 0.38	0.26^*^	9.42 ± 0.4	9.56 ± 0.35	0.22^**^
Vitamin D *(ng/mL)*	33.3 ± 11.78	32.19 ± 10.82	36.3 ± 14.26	0.37^*^	34.65 ± 12.93	32.15 ± 10.93	0.37^*^	32.00 ± 9.39	35.69 ± 15.43	0.37^*^

*Independent sample t test, ^**^Mann-Whitney

SD: Standard Deviation, MMSE: Mini-Mental State Examination, MoCA: Montreal Cognitive Assessment, SDLT: Serial Digit Learning Test BACE1: Beta-Site Amyloid Precursor Protein Cleaving Enzyme1, IL-6: Interleukin-6, MDA: Malondialdehyde, NO: Nitric Oxide, TAC: Total Antioxidant Capacity, RF: Rheumatoid Factor, CRP: C-Reactive Protein, ESR: Erythrocyte Sedimentation Rate, WBC: White Blood Cell, RBC: Red Blood Cell, BUN: Blood Urea Nitrogen, FBS: Fasting Blood Sugar, HDL: High-Density Lipoprotein, LDL: Low-Density Lipoprotein, PLT: Platelet Count

Covariates Adjustments include the following: Cognitive assessments; MMSE, MoCA, SDLT: Adjusted for age, education, medication use (e.g., MTX, Prednisolone, and HCQ); Sleep quality: Adjusted for medication use (e.g., Benzodiazepine, Psychiatric Drugs, MTX, Prednisolone, and HCQ). Inflammatory Markers (ESR, CRP, RF): Adjusted for medication use (e.g., MTX, Prednisolone, and HCQ). Clinical Tests; Total Cholesterol: Adjusted for medication use (e.g., Atorvastatin, MTX, Prednisolone, and HCQ); Calcium: Adjusted for medication use (e.g., any type of Calcium supplements, MTX, Prednisolone, and HCQ); Vitamin D: Adjusted for medication use (e.g., any type of vitamin D supplements, MTX, Prednisolone, and HCQ)

### 3.7. Correlation of clinical, serological, and neurocognitive parameters

**Fig 2:**
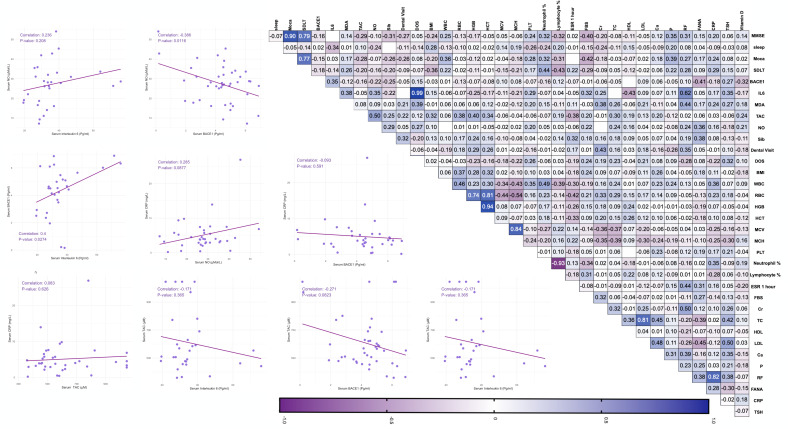
Correlogram and Scatter Plots of Sjogren Syndrome Patients. MMSE: Mini-Mental State Examination, MoCA: Montreal Cognitive Assessment, Sleep: Pittsburgh Sleep Quality Index, SDLT: Serial Digit Learning Test, BACE1: Beta-Site Amyloid Precursor Protein Cleaving Enzyme1, IL-6: Interleukin-6, MDA: Malondialdehyde, NO: Nitric Oxide, TAC: Total Antioxidant Capacity, FANA: Florescent anti-nucleotide antibody, WBC: White Blood Cell, RBC: Red Blood Cell, TSH: Thyroid Stimulating Hormone, FBS: Fasting Blood Sugar, Ca: Calcium, P: Phosphorus, HDL: High-Density Lipoprotein, LDL: Low-Density Lipoprotein, TC: Total Cholesterol, PLT: Platelet Count, Hgb: Hemoglobulin, Hct: Hematocrit, MCV: Mean Corpuscular Volume, MCH: Mean Corpuscular Hemoglobin, Cr: Creatinine, Sib: Number of Siblings, ESR: Erythrocyte Sedimentation Rate, BMI: Body Mass Index, DOS: Duration of Symptoms.

As part of the analysis clinical, serological, and neurocognitive assessments of Sjogren’s patients were illustrated in correlogram and scatter plots in Fig 2. Firstly, a Correlogram of Sjogren’s patients revealed several significant associations among BACE1 activity, inflammatory blood biomarkers, and neuropsychological testing parameters in SS patients. As we tend to report data only with significance, among cognitive performance measures, MMSE showed strong positive correlations with MoCA (r = 0.90, P-value < 0.001) and SDLT (r = 0.79, P-value < 0.001), highlighting their shared cognitive domains. Additionally, MMSE showed a weaker but statistically significant negative correlation with FBS (r = −0.4, P-value = 0.012) and a positive correlation with serum phosphorus (r = 0.35, P-value = 0.031). **Correlogram and Scatter Plots of Sjogren Syndrome Patients.**

MoCA demonstrated significant correlations with various inflammatory and metabolic parameters. It was positively correlated with WBC count (r = 0.36, P-value = 0.017), neutrophil percentage (r = 0.32, P-value = 0.044), and patient’s serum phosphorus levels (r = 0.39 P-value = 0.016). However, MoCA was inversely associated with FBS (r = −0.42, P-value = 0.007).

SDLT was significantly linked to inflammatory and metabolic parameters, including lymphocyte percentage (r = −0.43, P-value = 0.005), and neutrophil percentage (r = 0.44, P-value = 0.005). These associations highlight the interplay between systemic inflammation and cognitive function.

Regarding inflammatory biomarkers, serum IL-6 demonstrated significant positive correlations with BMI (r = 0.15, P-value < 0.001), and MDA levels (r = 0.38, P-value = 0.041). Interestingly IL-6 serum levels were strongly correlated with the duration of symptoms (r = 0.99, P-value < 0.001). Moreover, IL-6 showed a negative correlation with HDL cholesterol (r = −0.43, P-value = 0.030).

RF titer showed strong and significant positive correlation with CRP (r = 0.82, P-value < 0.001). Other significant correlations were observed among biomarkers of inflammation and oxidative stress. MDA showed positive associations with duration of symptoms (r = 0.39, P-value = 0.009), and Cr (r = 0.38, P-value = 0.015). TAC demonstrated a correlation with ESR (r = −0.38, P-value = 0.021) and NO (r = 0.5, P-value < 0.001). Moreover, TAC was significantly linked to several hematological markers, HCT (r = 0.34, P-value = 0.030), HGB (r = 0.4, P-value = 0.009), and RBC count (r = 0.38, P-value = 0.015).

Lastly, as expected LDL cholesterol correlates positively with total cholesterol (r = 0.81, P-value < 0.001). Serum calcium displayed significant associations with total cholesterol (r = 0.45, P-value = 0.005), LDL cholesterol (r = 0.48, P-value = 0.003), and TSH (r = 0.35, P-value = 0.044). Similarly, TSH was linked to LDL cholesterol (r = 0.5, P-value = 0.002).

Among Pearson analysis in scatter plots, BACE1 was positively correlated with IL-6 level (r = 0.4, P-value = 0.027) and negatively correlated with NO level (r = −0.386, P-value = 0.012).

## 4. Discussion

This study elucidated the critical role of oxidative stress and inflammatory factors, as well as BACE1, as a novel factor previously measured in AD diagnosis and treatment through the assessment of their blood levels in patients with SS and their potential role in the progression of neurocognitive symptoms in Sjogren’s syndrome patients.

SS, among other rheumatological diseases, has consistently shown a complex prognosis and relationship with various systemic involvements in the human body. It is widely recognized that the PNS and CNS are involved in SS and are frequently seen in clinical practice; however, the hypothesized mechanisms of action remain unclear and need further investigation [20]. As a general principle, chronic inflammatory diseases can influence CNS through their cytokines, inflammatory factors, and Reactive oxygen species (ROS) [21,22]. Nevertheless, as indicated by the results of our study and limited previous findings, there must be a more direct and precise mechanism by which SS affects the CNS [5].

BACE1 exhibits activity in the majority of human body cells and tissues; however, its maximal activity is observed in neuronal tissues and cells [23,24]. It’s a crucial enzyme in the amyloidogenic pathway, which cleavages amyloid precursor protein (APP), resulting in the production of amyloid-beta (Aβ), a key component in AD pathology [25]. Several human studies conducted on patients with cognitive impairments, including AD, have found that high BACE1 concentrations and activity in the CSF, serum, or plasma are associated with impairments in cognitive functions, as well as neuroimaging features, and brain changes in AD [26–32]. However, some studies have also reported no association between these factors [33,34].

Several studies have observed elevated BACE1 serum levels in individuals with early-stage AD who exhibited a better cognitive function and subsequently progressed to dementia, indicating the potential of serum BACE1 levels as an early predictor of cognitive decline [31,35,36]. Furthermore, certain studies have observed that patients in the late stages of dementia showed lower levels of BACE1, which may be attributed to brain atrophy and the loss of the synaptic sites [37].

Consequently, BACE1 has been considered an effective diagnostic tool for the detection of early AD symptoms and the development of neurocognitive diseases. Furthermore, recent clinical trial studies based on BACE1 inhibition as a sufficient approach for the prevention of AD are ongoing [5,38,39]. Although in our study, BACE1 levels in SS patients were not significantly different from the control group, this novel parameter was evaluated for the first time in SS and a limited number of times in rheumatological diseases. This finding suggests that the pathophysiology underlying cognitive decline in SS patients may differ from the key pathogenesis of AD, which is Aβ production.

The MMSE and MoCA are the most frequently utilized assessments for detecting declines in neurocognitive functions, particularly in the early phases such as mild cognitive impairment (MCI). As expected, these two scales showed a strong correlation; however, it has been observed in studies that social variables such as age and education can affect MMSE scores [40,41]. MoCA is often employed in individuals with cognitive decline who typically exhibit normal MMSE scores [42]. Furthermore, it has been reported that MoCA is superior to MMSE as a cognitive assessment tool and in the detection of early stages of cognitive impairments [43]. This can justify why we observed a significant decline in MoCA, and SDLT scores of patients with SS compared to controls, whereas their MMSE scores were lower but not significant.

Our findings suggest a potential role of IL-6 in the interplay between neuroinflammation and BACE1 levels, although this pathway was not to be altered in SS cognitive impairment. IL-6, a key proinflammatory cytokine, has been implicated in the pathogenesis of neurodegenerative diseases, such as MCI and AD [44–46]. In addition to the neuroinflammation effects of IL-6, in chronic diseases persistent expression of IL-6, is hypothesized to play a role in neurodegenerative processes by impacting pathways such as BACE1 regulation. Evidence indicates that nuclear factor-kappa B (NF-κB), a transcription factor activated during inflammation, can regulate BACE1 transcription, thereby enhancing Aβ production and exacerbating AD pathology [47,48]. Although NF-κB binding sites in the BACE1 promoter suggest regulatory mechanisms under inflammatory conditions, compelling evidence remains limited. Studies also indicate that increased IL-6 levels correlate with a higher likelihood of MCI progressing to AD [49,50]. However, variability in IL-6 levels over different disease stages, including undetectable levels in advanced AD, suggests that its role may be stage-specific [46]. The relationship between IL-6, BACE1, and cognitive decline underscores the central role of neuroinflammation in disease progression.

Additionally, our study revealed that IL-6 has been strongly correlated with symptom duration, supporting its potential use as a biomarker in tracking disease progression. This finding aligns with previous studies in rheumatological diseases. For instance, it has been reported that high serum IL-6 levels are associated with disease activity in rheumatoid arthritis (RA) and systemic lupus erythematosus, and with more severe clinical manifestations in scleroderma, indicating that IL-6 levels could serve as a biomarker for disease duration [51–55].

Our study results demonstrated higher FBS and lower serum phosphorus levels are commonly observed in patients with autoimmune diseases receiving immunosuppressive medications. Firstly, chronic inflammatory states can lead to insulin resistance and impaired glucose metabolism. Secondly, Corticosteroids are known to induce hyperglycemia by increasing gluconeogenesis and decreasing glucose uptake in peripheral tissues [56]. Our findings in this regard are consistent with other studies that reported similar observations in patients frequently receiving corticosteroids to manage their symptoms and disease activity, such as those with RA and SS [57,58]. Moreover, corticosteroids can impact mineral metabolism, leading to decreased absorption of phosphorus in the gastrointestinal tract and increased renal excretion [59]. In our study, FBS was also negatively correlated with neurocognitive tests, and phosphorus was positively correlated with MMSE and MoCA scores, suggesting that FBS and phosphorus levels may also affect cognitive function. It has been reported that elevated blood glucose levels can adversely impact cognitive performance, particularly attention, memory, and executive function [60,61]. Furthermore, phosphorus plays a critical role in neuronal function and energy metabolism; thus, lower phosphorus levels may also contribute to cognitive deficits by impairing synaptic function and neurotransmission [62].

It is also necessary to consider the possibility of other co-factors in this network, such as the long-term effect of medication therapy for SS. Adding complexity to this nexus of factors, a recent study indicated that recipients of HCQ showed reduced AD risk by enhancing the microglial clearance of Aβ1–42 and lowering the neuroinflammatory factors in mice [63]. This data contradicts a clinical trial study on HCQ in AD by Van Gool et al in 2001. This 18-month randomized; double-blind, placebo-controlled study demonstrated a significant effect of HCQ in reducing the rate of AD progression [64]. HCQ and Azathioprine have become commonly used standard medications for managing SS and other rheumatological disorders [65]. Azathioprine has also been reported to be involved in some neuropsychiatric presentations [66].

Moreover, Prednisolone, recognized as an effective and the most commonly administered for autoimmune disease, is also a significant factor in this network. A meta-analysis of 26 studies on corticosteroids suggested a mild positive effect of corticosteroids on memory and cognition [67]. Considering our results, we found no significant difference between treatment-positive and treatment-negative patients in serum inflammatory factors and BACE1 or neurocognitive tests for these three treatments. However, as these drugs are administered in immunosuppressive doses, they showed their expected effect on WBC, which was lower in all three medication groups (P-value = 0.03). This indicates the efficiency of the treatment and other results can be better trusted as these expected results occurred.

Another aspect to consider in this study is the reported data on the number of siblings. This consideration stems from the hygiene hypothesis, which indicates that small-sized families with fewer siblings may be at risk of autoimmune disease due to reduced exposure to allergens and infectious microorganisms [68]. However, we did not find any significant result to confirm this theory at any point in our results.

## 5. Conclusion

As SS patients demonstrated significantly lower performance in MoCA and SDLT questionnaires, BACE1 and inflammatory markers such as IL6, MDA, TAC, and NO were not significantly altered in SS. The MMSE test is highly dependent on education level and does not adjust the scores based on education, unlike MoCA. The PSQI test was not statistically altered in SS. This indicates a cognitive decline in SS is present, but the underlying mechanism still needs further investigation. Medication therapy with DMARDs and Prednisolone did not alter neurocognitive and inflammatory factors. Some noteworthy findings were observed in SS, including significantly higher dental visits, as well as elevated RF and monocyte percentage, and lower WBC count, LDL, and calcium levels, despite significant use of calcium supplements in SS patients, as expected.

Positive correlations observed between elevated IL-6 levels, as a key interleukin in the inflammation process and BACE1, suggest a potential relationship between inflammation and BACE1, although this pathway was not altered in SS cognitive impairment. Furthermore, IL-6 demonstrated a strong correlation with the duration of symptoms, potentially serving as a practical marker in tracking the disease progress. Additionally, as higher FBS and lower serum phosphorus are commonly observed in patients with autoimmune diseases receiving immunosuppressive medications, FBS showed a negative correlation with neurocognitive tests, and phosphorus exhibited a positive correlation with MMSE and MoCA scores.

## 6. Limitations and strengths

Our study executes a comprehensive result of hematological and neuropsychological testing on 81 participants. The relatively limited sample size of this study should be noted and future studies in this field with more populated sample size is recommended. To our knowledge, BACE1 is measured for the first time in SS and any other rheumatological disease to assess the theory of Aβ production in SS. This provides a new insight into SS, as the neurological manifestations of this disease have not been adequately addressed. Examining all three aspects of a disease (neuropsychological and demographic assessment, biomarker and blood test, signs and symptoms of disease data) could enhance our understanding of the SS effect on the CNS by incorporating all the factors into the analysis. MDA, TAC, NO, and IL-6 measurements of the SS patients as key inflammatory tests allowed us to better understand this complex interplay and determine if they play a role in the inflammatory mechanism of the SS pathophysiology, BACE1 level, or Aβ production.

There are some limitations of this study that need to be addressed. For a better understanding of the Aβ production pathway in SS, it is recommended that future studies assess the Aβ1–42 and Aβ1–40 in their studies, as well as tau protein and other related molecular tests to better elucidate this pathway. Additional pathways should be considered in the CNS involvement of SS. While we measured IL-6 and MDA as proposed mechanisms of inflammation and Aβ production, we were unable to assess alternative mechanisms in which we might observe significance. Lastly, as our control and SS participants were not all subjectable to imaging, we were not able to include a comprehensive assessment of imaging. Future studies could benefit from imaging techniques to better physically assess the effect of SS on the brain.

## Supporting information

S1 Data Dataset. (XLSX)
